# STay tunEd: mutational analysis of the *HvSTE1* gene in barley provides insight into the balance between semi-dwarfism and maintenance of grain size in brassinosteroid biosynthesis-dependent manner

**DOI:** 10.3389/fpls.2025.1571368

**Published:** 2025-05-06

**Authors:** Karolina Zolkiewicz, Jana Oklestkova, Beata Chmielewska, Damian Gruszka

**Affiliations:** ^1^ Institute of Biology, Biotechnology and Environmental Protection, Faculty of Natural Sciences, University of Silesia, Katowice, Poland; ^2^ Laboratory of Growth Regulators, Faculty of Science, Palacký University and Institute of Experimental Botany, Czech Academy of Sciences, Olomouc, Czechia

**Keywords:** barley, biosynthesis, brassinosteroids, plant architecture, plant reproduction, TILLING, grain size

## Abstract

Brassinosteroids (BRs) are steroid phytohormones which regulate various physiological and developmental processes throughout plant life cycle. The BR biosynthesis has been studied mainly in the dicot model species - *Arabidopsis thaliana.* However, our current understanding of the BR biosynthesis and its regulation in other species, including cereal crops, is limited. Functions of enzymes which catalyze early stages of the BR biosynthesis in cereals remain poorly understood. Moreover, mechanisms regulating expression of genes encoding these enzymes remain obscure. One of the genes which participate in the early stages of the BR biosynthesis in Arabidopsis is *STE1* (*STEROL DESATURASE1*). However, detailed functional analyses of this gene and its promoter region have not been performed. The aim of this study was to identify and functionally analyze the *STE1* gene in barley (*Hordeum vulgare*) which is an important cereal crop. The functional analysis was carried out with the application of TILLING (Targeting Induced Local Lesions IN Genomes) approach. Six mutations were identified within the 1st exon (including three located in the 5’UTR region) and one missense mutation was identified in the 2nd exon of *HvSTE1.* Effects of the identified alleles on the *HvSTE1* gene expression, sequence of the encoded enzyme variants, BR accumulation, as well as on stature, agronomic traits, and reproduction of the identified mutants were characterized. Homozygous mutants carrying two alleles (*hvste1.b* and *hvste1.o*) displayed reduced plant height and defects in the BR accumulation. The *HvSTE1* expression was considerably decreased in the 3rd internode of the *hvste1.b* mutant. Interestingly, the *hvste1.b* mutant plants showed semi-dwarf phenotype without any negative effect on crucial agronomic traits, such as tiller number, spike length, and grain weight. Moreover, weight of grains produced by the *hvste1.b* mutant was slightly (5%) higher when compared with the reference cultivar. The results of this study provided a novel insight into the function of the *HvSTE1* gene in the BR biosynthesis-dependent regulation of architecture and reproduction of barley. Moreover, the *hvste1.b* allele allows for achieving a balance between the favorable alteration in plant architecture (semi-dwarfism) and maintenance (slight improvement) of grain weight in this species.

## 1 Introduction

Plant development and physiology are regulated by various phytohormones. Brassinosteroids (BRs) constitute a class of steroid phytohormones which were identified in the 1970’s (Mitchell et al., 1970; Grove et al., 1979). Research conducted since the time of the BR discovery and based on analysis of BR-deficient and BR-insensitive mutants indicated that BRs regulate various physiological and developmental processes in plants, throughout their life cycle (Vriet et al., 2012; Tong and Chu, 2018; Nolan et al., 2020). Importantly, when compared with other phytohormones, BRs are exceptional, as they do not undergo a long-distance transport between plant organs (Symons et al., 2008). Only relatively recently an insight into the BR export from cell and short-distance (cell-to-cell) BR transport has been gotten (Vukasinovic and Russinova, 2018; Wang et al., 2023; Ahmar and Gruszka, 2024; Ying et al., 2024). Noteworthy, it is known that BRs are accumulated in all plant tissues and organs, however, at various concentrations, depending on plant species, tissue, and developmental stage (Clouse and Sasse, 1998; Kanwar et al., 2017).

As far as the BR biosynthesis process is concerned, it has been studied with the use of various approaches for more than two decades mainly in the dicot model species - *Arabidopsis thaliana* (Shimada et al., 2001; Fujioka and Yokota, 2003; Vriet et al., 2012; Chung and Choe, 2013; Bajguz et al., 2020). In a broader context, the BR biosynthesis is a part of the process of sterol biosynthesis (Lindsey et al., 2003; Bajguz et al., 2020). Noteworthy, the BR biosynthesis is a complicated process, and its numerous biochemical reactions are catalyzed by different enzymes (Bajguz et al., 2020). However, our current understanding of the BR biosynthesis and its regulation in other species, including cereal crops, is rather limited when compared with Arabidopsis (Gruszka, 2019). Nevertheless, several genes involved in the BR biosynthesis were functionally characterized also in cereals, such as rice (*Oryza sativa*) (Hong et al., 2002, 2005; Sakamoto et al., 2006, 2012; Li et al., 2018; Zhan et al., 2022), maize (*Zea mays*) (Tao et al., 2004; Liu et al., 2007; Hartwig et al., 2011; Makarevitch et al., 2012; Sun et al., 2021), and barley (*Hordeum vulgare*) (Gruszka et al., 2011; Dockter et al., 2014; Gruszka et al., 2016a). However, although the first insight into some of the enzymatic steps of the BR biosynthesis (functioning in the downstream part of this pathway) has been obtained in these cereals, it should be kept in mind that our current knowledge about the function of enzymes which catalyze reactions at the junction of the sterol and BR biosynthesis pathways (i.e. early stages of the BR biosynthesis) remains very limited, even in the cereal model species - rice. Moreover, the current knowledge about mechanisms regulating expression of genes encoding these enzymes remains very limited as well. Therefore, further studies in these research areas are required.

One of the genes which are known to participate in the sterol biosynthesis and the early stages of the BR biosynthesis in Arabidopsis is *STE1* (*STEROL DESATURASE1*), also known as *DWARF7*, which encodes Δ(7)-Sterol-C5-desaturase catalyzing the biosynthesis of 5-dehydroavenasterol during the sterol biosynthesis and 5-dehydroepisterol during the early stages of the BR biosynthesis (Choe et al., 1999; Taton et al., 2000; Lindsey et al., 2003; Bajguz et al., 2020). It was reported that two nonsense mutations located in different exons of the *STE1* gene resulted in a very significant plant height reduction in the isolated Arabidopsis mutants (the mutant plants reached only ca. 14% height of the wild-type plants), abnormalities in positions of vascular bundles, reduced fertility manifested by 4-fold decrease in the number of produced seeds, and prolonged life span (Choe et al., 1999; Husselstein et al., 1999). The dwarf phenotype of the isolated mutants was chiefly caused by defects in cell elongation (Catterou et al., 2001). Although it was shown that the *STE1* gene plays the important role in regulation of growth and reproduction in Arabidopsis, a detailed functional analysis of this gene (i.e. effects of substitutions of individual amino acid residues in the encoded enzyme) has never been performed, even in this model species. Analysis of promoter region of the *STE1* gene has never been carried out either.

As it was mentioned above, the functional (mutational) analysis of a homologue of the *STE1* gene has never been performed in any cereal species. However, it should be kept in mind that in cereals the plant height reduction (semi-dwarfism) is of particular importance. Noteworthy, cereal mutants defective in the BR biosynthesis show semi-dwarfism and erect stature (Tanabe et al., 2005; Sakamoto et al., 2006; Dockter et al., 2014; Best et al., 2016). These phenotypic traits are very important in cereal breeding, as in contrast to tall cereal varieties, the semi-dwarf, erect plants are more tolerant to lodging which occurs during unfavorable weather conditions and constitutes a serious threat to plant reproduction and yield. Thus, several semi-dwarf mutants (including BR-related mutants) have already proven very valuable in cereal breeding (Chono et al, 2003; Dockter and Hansson, 2015) and it has been suggested that the semi-dwarf, cereal BR mutants may constitute an alternative in the future breeding programs due to their erect stature (allowing dense planting), improved tolerance to environmental stresses, and enhanced nitrogen-use efficiency (Sakamoto et al., 2006; Dockter et al., 2014; Gruszka et al., 2016b; Ahmar and Gruszka, 2023; Song et al., 2023). Therefore, characterization of the BR biosynthesis-dependent mechanisms of regulation of plant architecture, development, reproduction, and yield is of crucial importance in cereals. However, during the optimization of cereal plant stature a balance between plant growth reduction (semi-dwarfism) and maintenance of plant yield needs to be retained. Noteworthy, several cereal mutants defective in the BR biosynthesis (due to loss-of-function mutation or gene downregulation) show reduction in yield as a side effect (Hong et al., 2005; Makarevitch et al., 2012; Wu et al., 2016; Zhou et al., 2017; Xu et al., 2022; Zhang et al., 2024). Taking it into account, the aim of the current research should be development of the BR-deficient mutants with the semi-dwarf, erect stature, which is regarded as the favorable trait in cereal cultivation (Sakamoto et al., 2006; Ahmar and Gruszka, 2023), however, without the negative effect on plant yield.

Therefore, the aim of this study was to identify and functionally analyze the *STE1* gene (including its promoter region) in barley (*Hordeum vulgare*) which is an important cereal crop. The functional analysis was carried out with the application of TILLING (Targeting Induced Local Lesions IN Genomes) approach. This study led to identification of seven mutations located in promoter region and coding sequence of this gene. Effects of the identified alleles on the *HvSTE1* gene expression, sequence of the encoded enzyme variants, accumulation of the bioactive form of BR - brassinolide, as well as on stature, agronomic traits, and reproduction of the identified mutants were characterized. Therefore, the results of this study provided a novel insight into the function of the *HvSTE1* gene in the BR biosynthesis-dependent regulation of architecture and reproduction of barley.

## 2 Materials and methods

### 2.1 Plant material

**Figure 1:**
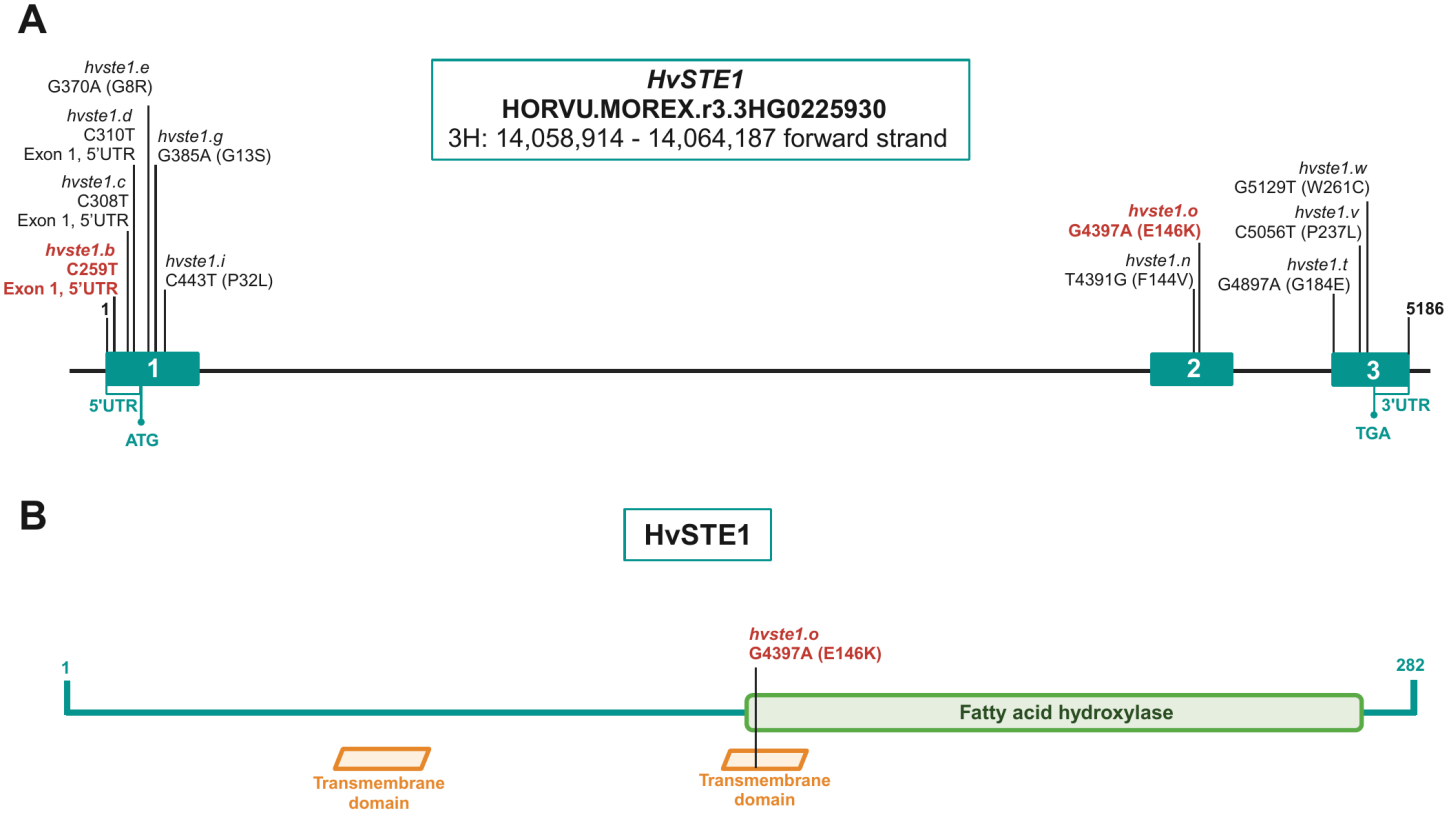
**(A)** The *HvSTE1* gene structure with highlighted positions of the identified mutations within exons (blue rectangles). Mutations which resulted in the reduction of plant height in the analyzed mutants are shown in red color. **(B)** Localization of the amino acid residue (E146), substituted as a result of the *hvste1.o* mutation, within the structural (transmembrane) and functional (fatty acid hydroxylase) domains of the HvSTE1 protein.

The plant material for this study comprised barley (*Hordeum vulgare*) mutants from the HorTILLUS population, developed through the TILLING approach at the Department of Plant Genetics and Functional Genomics (University of Silesia, Poland), and the reference cultivar ‘Sebastian’. The identified mutants contain mutations within the 1st exon (alleles *hvste1.b, hvste1.c, hvste1.d, hvste1.e, hvste1.g, hvste1.i*) and the 2nd exon (allele *hvste1.o*) of the *HvSTE1* gene HORVU.MOREX.r3.3HG0225930 (**Figure 1**; **Table 1**). The HorTILLUS mutant population, its development, and the TILLING method were broadly described in our previous article (Szurman-Zubrzycka et al., 2018). In this study, homozygous mutant lines were applied for all identified alleles. In order to fix homozygosity of the mutant lines, the experiments were carried out on the homozygous mutant lines from the M_4_ or M_5_ generation. In each generation from M_3_ to M_5_, phenotypes of the homozygous mutants were monitored and analyzed. In case of each of the analyzed alleles and the homozygous mutant lines, the phenotypes remained stable from generation to generation (up to M_4_ or M_5_). For all analyzed alleles several homozygous mutant lines were identified and included in this study. The homozygous mutant lines representing specific allele exhibited the same phenotype. This confirms that the mutations analyzed in this study are responsible for the mutant phenotypes.

**Table 1:** The identified mutations within the *HvSTE1* gene and their impact on the sequence of encoded protein or gene expression, as well as on the phenotype (height) of the mutants.

Allele	Position of mutation	Effect of mutation	Phenotype
***hvste1.b***	**C259T (5’UTR)**	**Alteration in gene expression**	**Semi-dwarf**
*hvste1.c*	C308T (5’UTR)	–	Normal
*hvste1.d*	C310T (5’UTR)	–	Normal
*hvste1.e*	G370A (Exon 1)	G8R	Normal
*hvste1.g*	G385A (Exon 1)	G13S	Normal
*hvste1.i*	C443T (Exon 1)	P32L	Normal
***hvste1.o***	**G4397A (Exon 2)**	**E146K**	**Dwarf**

Mutations which resulted in the plant height reduction are bolded.

### 2.2 Verification of mutations of the *HvSTE1* gene in homozygous mutant plants

In order to isolate homozygous barley mutant plants with mutations identified in the *HvSTE1* gene with the use of TILLING procedure, progeny of selected M_2_ plants from the HorTILLUS population were grown in a greenhouse under 16-hour photoperiod. Light intensity amounted to 400 µmol m^−2^ s^−1^. Plant DNA was isolated from leaves of 3-week-old seedlings of the homozygous mutants and the ‘Sebastian’ cultivar (reference) according to the REDExtract-N-Amp™ Plant PCR Kit (Sigma-Aldrich, Poole, Dorset, UK) protocol. The putative *HvSTE1* gene sequence (acc no. HORVU.MOREX.r3.3HG0225930) was retrieved from the Ensembl Plants database (https://plants.ensembl.org/index.html) using the *STE1* gene sequence from *Arabidopsis thaliana*, as well as encoded transcript and protein sequences as queries during the database search. Primers for PCR amplifications of the *HvSTE1* gene sequence and for Real Time quantitative PCR (RT-qPCR) analysis of the *HvSTE1* gene expression were designed using the Jellyfish software (LabVelocity, San Francisco, CA, USA). The primer sequences are available in **Supplementary Materials** along with the PCR amplification profiles (**Supplementary Tables S1**, **S2**). The PCR reactions were carried out using the Applied Biosystems™ SimpliAmp™ Thermal Cyclers. The obtained PCR products were sequenced (outsourced service provided by Genomed, Warsaw) and the sequencing data were analyzed using licensed CodoneCode Aligner tool.

### 2.3 Measurement of endogenous brassinolide concentration

Fragments of leaf tissues were collected from the second (developmentally) leaves when plants of the analyzed genotypes developed two or three tillers (at the same developmental stage). The leaf fragments (1 g F.W. per sample) were collected from the first (developmentally) tiller. Each sample contained 3 leaf fragments collected from 3 individual plants (polled together) per 1 biological replicate. In each genotype, the leaf sampling was performed in 3 biological replicates. Thus, in each genotype the leaf fragments were collected from 9 plants. The extraction procedure and quantification of endogenous brassinolide (BL) with the use of Ultra High Performance Liquid Chromatography (UHPLC-MS/MS) were described in detail in our previous article (Gruszka et al., 2016b). Each measurement was performed in three repetitions.

### 2.4 RNA isolation and gene expression analysis

The Ensembl Plants (https://plants.ensembl.org/index.html) (Yates et al., 2022), EoRNA (https://ics.hutton.ac.uk/eorna/index.html) (Milne et al., 2021), and ePlant (https://bar.utoronto.ca/eplant_barley/) databases were searched in order to obtain information about the *HvSTE1* transcript variants and their expression profiles. To specify the abundance of particular transcripts in the analyzed tissues, only experiments in which barley plants were not subjected to any stress conditions were taken into account. The RNA extraction followed by purification was performed from mature embryos and the 3rd internodes of developing tillers at six leaf stage of the homozygous mutant plants and the ‘Sebastian’ cultivar (reference) according to the TRIpure Reagent protocol (Total RNA Extraction Kit, Roche). Reverse transcription was carried out according to the protocol for NG dART RT kit (EURx, Poland) and RevertAid First Strand cDNA Synthesis Kit (Thermo Scientific), preceded by the RNA treatment with RNase-free DNase (Promega) according to the manufacturers’ protocols. The RT-qPCR analysis was performed with the use of LightCycler 480 II instrument (Roche) with the use of primers and the reaction profile shown in **Supplementary Tables S3** and **S4**. In this experiment two housekeeping genes, *Elongation Factor 1-a* (*HvEF1*; Gene Bank Acc. No. AJ472912) and *Histone H2A* (*HvH2A*; Gene Bank Acc. No. AK251274.1) were analyzed as references (Chandna et al., 2012; Rapacz et al., 2012) to measure the relative expression level of the *HvSTE1* gene. The relative expression level of the *HvSTE1* gene was determined according to the comparative *C*_T_ method, also referred to as the 2^-ΔΔCT^ method, for quantitative gene expression studies (Schmittgen and Livak, 2008). The experiment was performed in two biological replicates. In each biological replicate, the RT-qPCR reactions were performed in three technical replicates for each of the analyzed genes.

### 2.5 *In silico* analysis of the *STE1* regulatory region, sequence and structure of the encoded protein and impacts of the identified mutations

The promoter sequences of homologous *STE1* genes from 6 species (*Arabidopsis thaliana*, *Hordeum vulgare, Helianthus annuus, Solanum lycopersicum, Oryza sativa, Brachypodium distachyon*) were retrieved from the EnsemblPlants database (https://plants.ensembl.org/index.html). To analyze the conserved motifs within the promoter regions the MEME tool (http://meme-suite.org/index.html) (Bailey et al., 2009) was used, whereas the TBtools v0.6655 toolkit (Chen et al., 2020) was applied for visualization. During the data submission for these analyses, the number of motifs was set at 10 per sequence. In order to determine Transcription Factor binding sites (TFbs) in the promoter regions, the PlantPAN 3.0 software (https://plantpan.itps.ncku.edu.tw/plantpan3/) was applied. With the use of Clustal Omega tool (https://www.ebi.ac.uk/Tools/msa/clustalo/) (Sievers et al., 2011) the multiple sequence alignment (MSA) of the STE1 proteins was generated based on 8 orthologs from the following dicot and monocot species: *Arabidopsis thaliana*, *Sorghum bicolor*, *Zea mays*, *Hordeum vulgare*, *Triticum aestivum, Aegilops tauschii*, *Brachypodium distachyon*, *Oryza sativa* which were retrieved from the NCBI (https://www.ncbi.nlm.nih.gov/) database. The impact of amino acid substitution on the activity of HvSTE1 protein was analyzed using the I-Mutant3.0 (https://folding.biofold.org/i-mutant/) and SIFT (https://sift.bii.a-star.edu.sg/) tools. The *HvSTE1* gene ontology was obtained with the use of ShinyGO v0.741 software (http://bioinformatics.sdstate.edu/go74/). The 3D structure of HvSTE1 protein was modeled with the use of AlphaFold Server (https://alphafoldserver.com/) and visualized using UCSF ChimeraX (https://www.cgl.ucsf.edu/chimerax/) (Pettersen et al., 2021).

### 2.6 Data visualization

Plots presenting phenotypic characterization of the *hvste1.b* and *hvste1.o* mutants and the reference cultivar ‘Sebastian’ were created with the use of R 4.4.0 software and the ggstatsplot package (Patil, 2021). The rest of the plots were constructed using the ggplot2 and gridExtra packages (Wickham, 2016; Auguie and Antonov, 2022).

### 2.7 Statistical analysis

All of the statistical calculations were carried out in STATISTICA version 12.0 (StatSoft Inc. 2014), PAST 4.03 or R 4.4.0 software. When the dataset had normal distribution, t-test was conducted to compare each of the analyzed mutants with the reference cultivar, with p<0.05 (*) and p<0.01 (**) considered to be significantly different. Otherwise, when dataset did not meet the requirement of normal distribution for the t-test, a non-parametric Mann-Whitney U test was performed [p<0.05 (*) and p<0.01 (**)]. During the analysis of grain morphology and weight, the grain length, width and thickness parameters were calculated on the basis of 50 grains (n=50), and 100-grain weight parameter was determined on the basis of 10 replicates (n=10).

## 3 Results

### 3.1 Characterization of phenotype of the *hvste1* mutants

**Figure 2:**
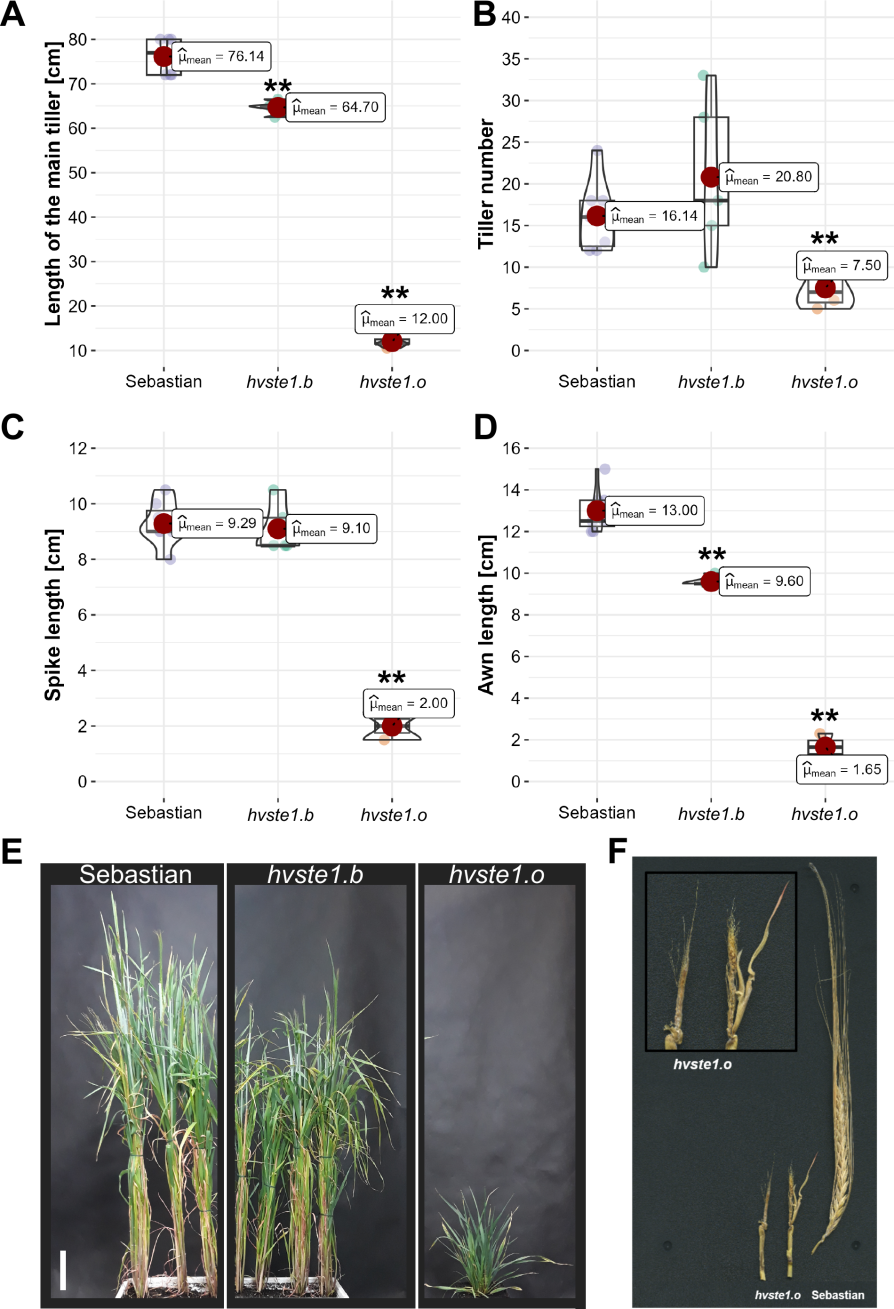
Phenotypic traits of the *hvste1.b* and *hvste1.o* mutants compared with the reference cultivar ‘Sebastian’ **(A-D)**. The measurements were performed on 10 plants of each genotype at the full maturity of plants (at harvest). Spike-producing tillers were taken into account when determining the tiller number. Asterisks represent level of significance (**indicates p<0.01). **(E)** Phenotypes of the *hvste1.b* and *hvste1.o* mutants in comparison to the reference cultivar ‘Sebastian’ plants (at the same developmental stage), scale bar: 10 cm. **(F)** Phenotype of the underdeveloped, sterile inflorescences produced by the *hvste1.o* mutant in comparison with fully developed spike of the ‘Sebastian’ cultivar.

**Figure 3:**
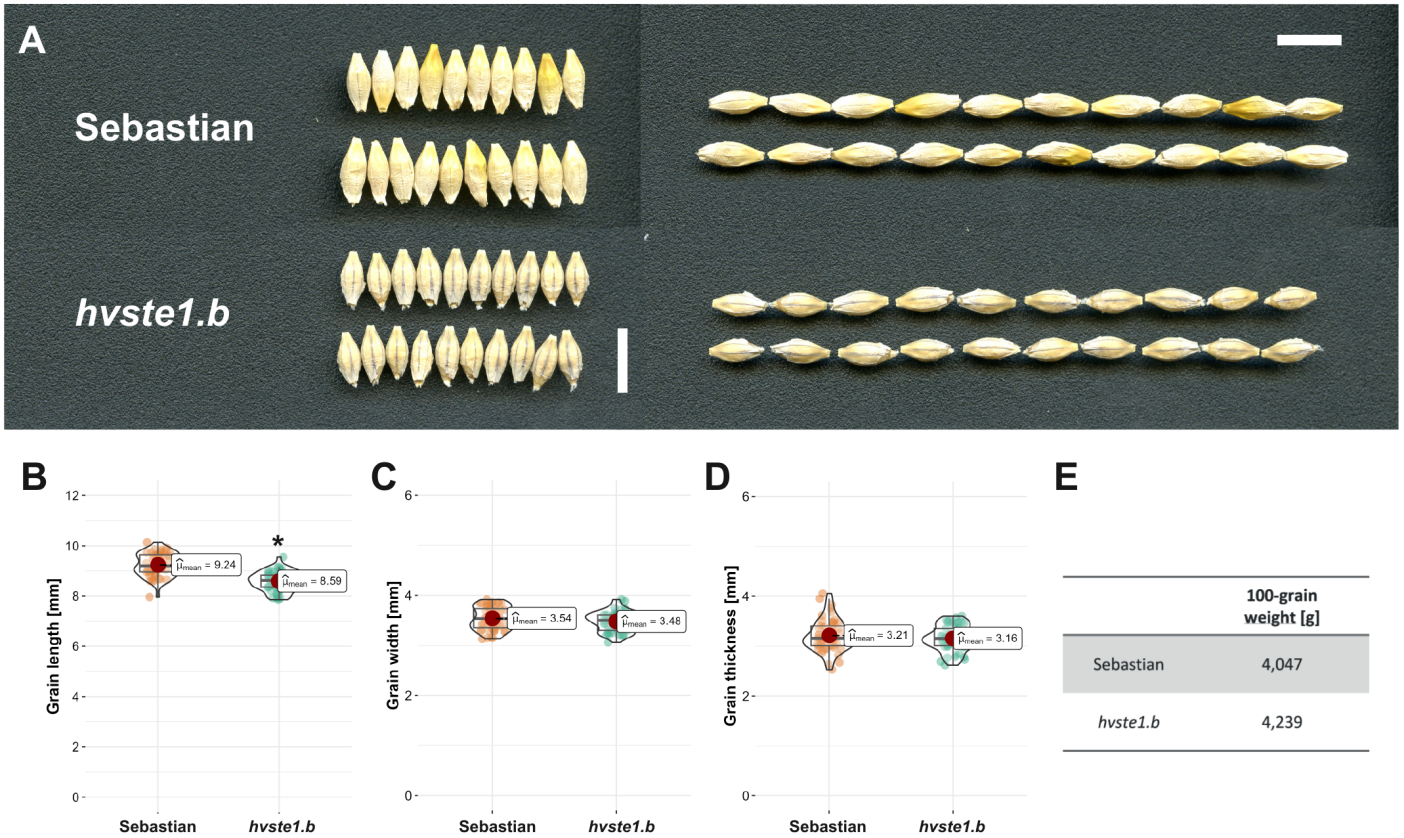
Phenotype of grains produced by the *hvste1.b* mutant in comparison with the respective phenotypes of the reference cultivar ‘Sebastian’. **(A)** Grain morphology of the *hvste1.b* mutant and the ‘Sebastian’ cultivar. Scale bar: 1 cm. **(B-E)** Grain length, width, thickness, and 100-grain weight of the *hvste1.b* mutant and the ‘Sebastian’ cultivar. The mean values are presented for each genotype, with error bars representing standard deviation. The grain length, width, and thickness parameters were calculated in each genotype on the basis of 50 grains (n=50) derived from 10 plants of this genotype, while 100-grain weight was determined on the basis of measurement of 10 batches of 10 grains, where 1 batch represented 1 plant of a given genotype; 10 replicates (n=10). Asterisks represent level of significance (*indicates p<0.05).

In order to get an insight into molecular function of the *HvSTE1* gene (HORVU.MOREX.r3.3HG0225930) in barley, a series of alleles was obtained with the use of TILLING approach. As a result of this strategy, six mutations were identified within the 1st exon (among which three were located in the 5’UTR region) and additionally one missense mutation was identified in the 2nd exon of *HvSTE1* (**Figure 1A**). Given the fact that homozygous mutant plants harboring two of the analyzed mutations (*hvste1.b* and *hvste1.o*) displayed reduced plant height (**Table 1**), these mutations were selected for further analyses. Considering the important role of BRs in the cereal crop development, including regulation of plant height, tiller number, and inflorescence architecture (Vriet et al., 2012; Dockter et al., 2014; Tong and Chu, 2018), these traits were analyzed in detail (**Figures 2A–D**). Phenotypes of the *hvste1.b* and *hvste1.o* mutants in comparison to the reference cultivar ‘Sebastian’ plants (at the same developmental stage) are shown in **Figure 2E**. Homozygous mutants carrying two of the analyzed mutations (alleles *hvste1.b* and *hvste1.o*) consistently exhibited significantly reduced plant height and awn length (**Figures 2A, D**). Interestingly, the *hvste1.b* mutant plants showed semi-dwarf phenotype without any negative effect on fertility. Therefore, apart from the overall plant phenotype, the agronomic traits related to grain size and weight were also measured (**Figures 3A–E**). Grains produced by the *hvste1.b* mutant plants were slightly shorter when compared with grains of the ‘Sebastian’ cultivar (**Figures 3A, B**). However, width and thickness of grains produced by the *hvste1.b* mutant did not significantly vary from the respective parameters reported in the ‘Sebastian’ cultivar (**Figures 3C, D**). Surprisingly, weight of grains produced by the *hvste1.b* mutant plants was slightly (5%), but not significantly, higher when compared with the grain weight in the ‘Sebastian’ cultivar (**Figure 3E**). Taking into account the semi-dwarf phenotype of the *hvste1.b* mutant without any negative effect on the crucial agronomic traits, such as tiller number, spike length (**Figures 2B, C**) and grain weight (**Figure 3E**), the *hvste1.b* allele proved to be of particular importance for further research, as it was previously reported that semi-dwarf cereal BR mutants defective in the BR biosynthesis produced smaller grains which resulted in yield decrease (see: Discussion). Therefore, the *hvste1.b* allele allows for achieving a balance between the favorable alteration in plant architecture (semi-dwarfism) and maintenance of grain weight.

On the other hand, the *hvste1.o* mutant displayed severe dwarf phenotype as the mutant plants reached only 16% of height of the reference cultivar ‘Sebastian’ (**Figures 2A, E**). The *hvste1.o* mutant plants produced also significantly lower number of tillers (**Figure 2B**). Moreover, the *hvste1.o* mutant plants were completely sterile, due to the fact that their inflorescences (both spikes and awns) were underdeveloped (**Figures 2C, D**). Phenotype of the underdeveloped inflorescences produced by the *hvste1.o* mutant is shown in **Figure 2F**.

### 3.2 Analysis of endogenous BR concentration

**Figure 4:**
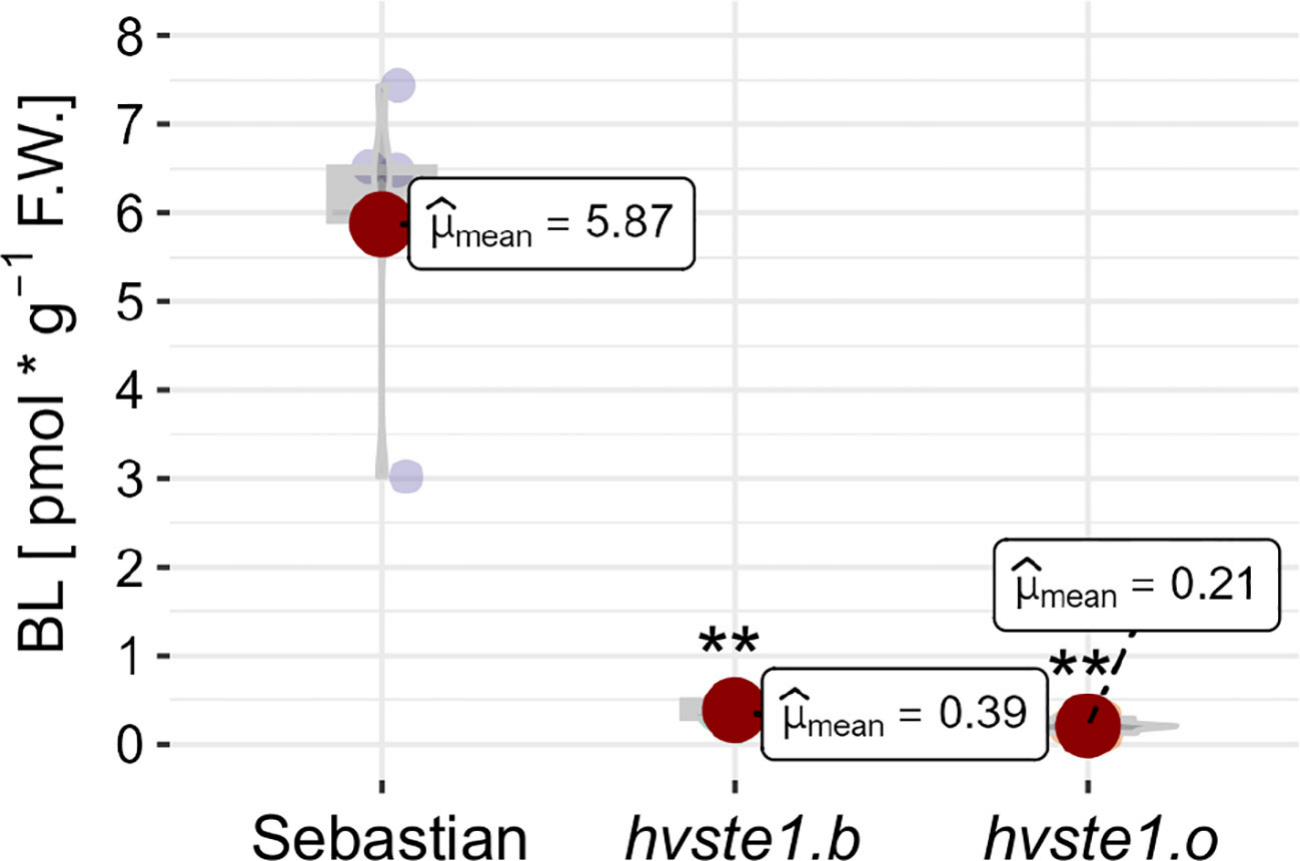
The endogenous contents of brassinolide (BL) in the analyzed genotypes. The mean values of three replicates of each measurement are presented for each genotype, with error bars representing standard deviation. Asterisks represent level of significance (**indicates p<0.01).

In order to verify the significant effect of the *hvste1.b* and *hvste1.o* mutations on the BR biosynthesis, the accumulation of biologically active brassinolide (BL), which is one of the final products of this process, was measured. In accordance with the above genetic and phenotypic results, the accumulation of BL was significantly lower in both of the analyzed mutants in comparison with the reference cultivar ‘Sebastian’ (**Figure 4**). These results demonstrate that both *hvste1.b* and *hvste1.o* cause an evident defect in the BR biosynthesis process.

### 3.3 Analysis of the *HvSTE1* transcription profile

**Figure 5:**
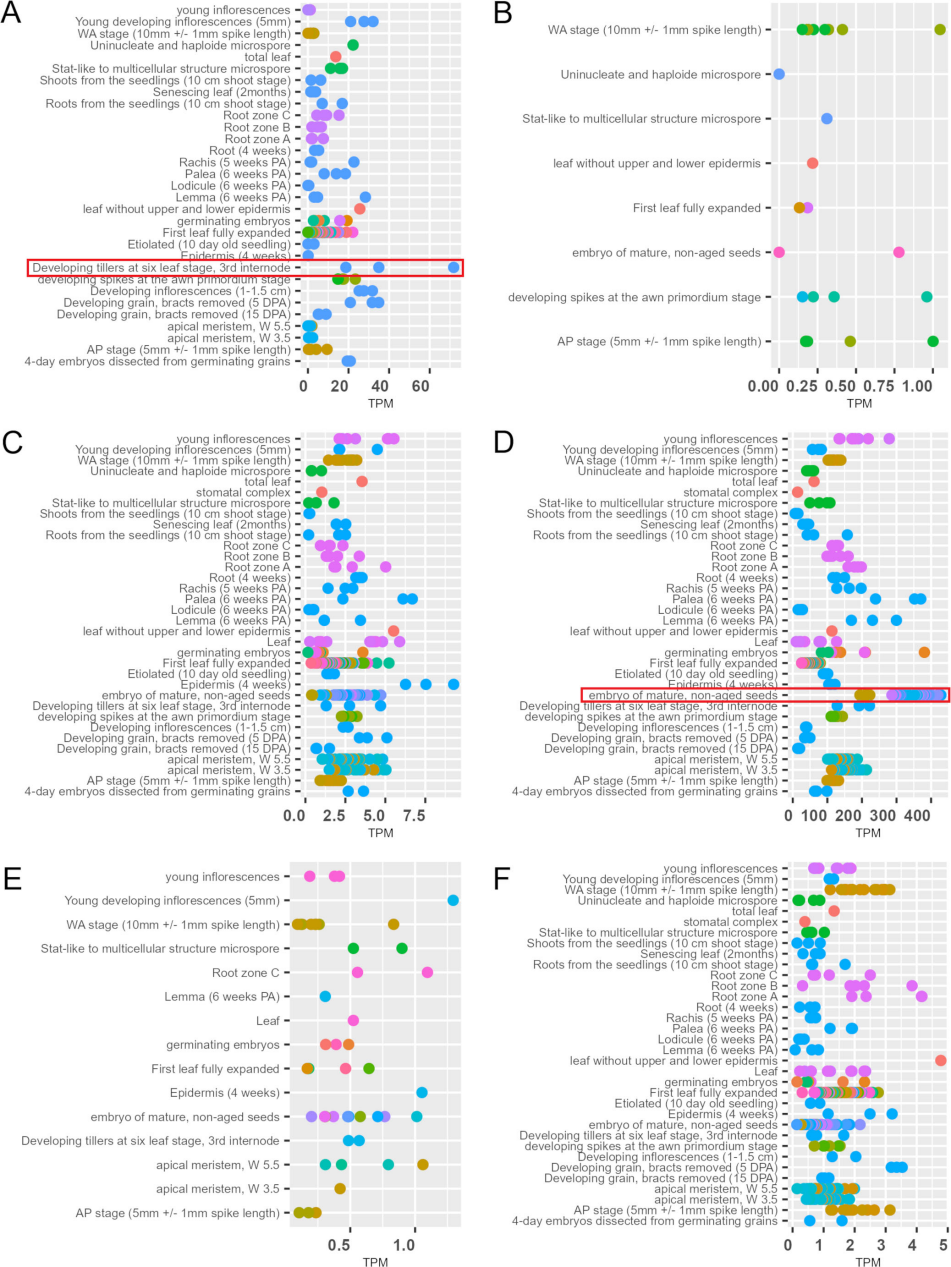
Tissue-specific expression of six *HvSTE1* transcript variants [**(A)** BART1_0-u17568.001, **(B)** BART1_0-u17568.002, **(C)** BART1_0-u17568.003, **(D)** BART1_0-u17568.004, **(E)** BART1_0-u17568.005, **(F)** BART1_0-u17568.006] in various tissues and organs of different barley cultivars (TPM, transcript per million). The expression data for these transcripts in various tissues were retrieved from the EoRNA database. Different colors represent various barley cultivars. Particularly high expression of the *HvSTE1* transcript variants observed in the 3rd internode of developing tillers at six leaf stage and embryos of mature, non-aged grains is highlighted with red frames. The whole figure with legends for each graph containing names of the barley cultivars is available in **Supplementary Materials** (**Supplementary Figure S2**).

**Figure 6:**
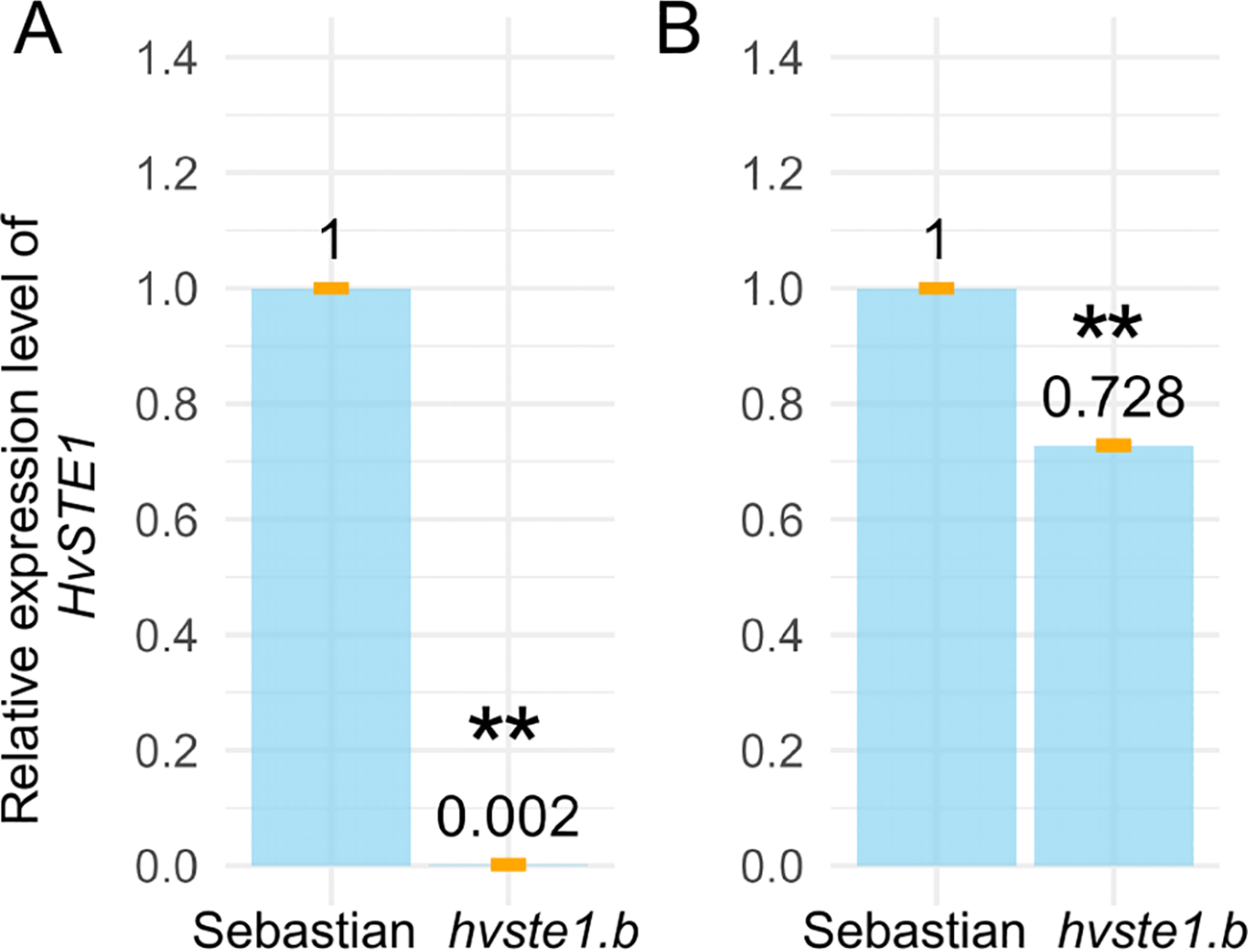
Transcription level of the *HvSTE1* gene in the 3rd internode **(A)** and embryos of mature grains **(B)** of the *hvste1.b* mutant and the reference cultivar ‘Sebastian’. Asterisks represent level of significance (**indicates p<0.01). Mean values are present with error bars (orange) representing standard deviation.

Given the fact that the *hvste1.b* mutation is located within the regulatory region of the *HvSTE1* gene (**Figure 1A**; **Table 1**) and the mutant plants show semi-dwarf phenotype and defects in the BR biosynthesis, the potential impact of this mutation on the gene expression was investigated. On the basis of information retrieved from the EoRNA database (https://ics.hutton.ac.uk/eorna/index.html), transcript encoded by the *HvSTE1* gene has six splice variants and each of them is characterized by different level of tissue-specific expression (**Figures 5A–F**). The *HvSTE1* gene expression data retrieved from the Barley Expression Database EoRNA and the Barley ePlant on the Bio-Analytic Resource (BAR) for Plant Biology database indicated that the highest average expression of various transcript variants of this gene had been reported in the 3rd internode of developing tillers at six leaf stage (**Figure 5A**) and in embryos of mature, non-aged grains (**Figure 5D**). Taking into account the data, these two tissues were analyzed using the RT-qPCR method in order to determine the relative expression of the *HvSTE1* gene in the *hvste1.b* mutant and the reference cultivar ‘Sebastian’. Importantly, the *HvSTE1* gene expression was considerably decreased, by 500-fold in the 3rd internode of the *hvste1.b* mutant in comparison with the ‘Sebastian’ cultivar (**Figure 6A**). It was also reported that the *HvSTE1* gene expression was relatively higher in the mature embryos of the *hvste1.b* mutant, however, the expression observed in the *hvste1.b* mutant was still lower (by ca. 27%) when compared with the reference cultivar (**Figure 6B**). These results indicated that the *hvste1.b* mutation caused a decrease in the *HvSTE1* gene expression, what was particularly manifested by the significantly diminished expression of this gene in the 3rd internode of the *hvste1.b* mutant plants.

### 3.4 Impact of the *hvste1.b* mutation on transcription factor-binding sites within regulatory region of the *HvSTE1* gene

**Figure 7:**
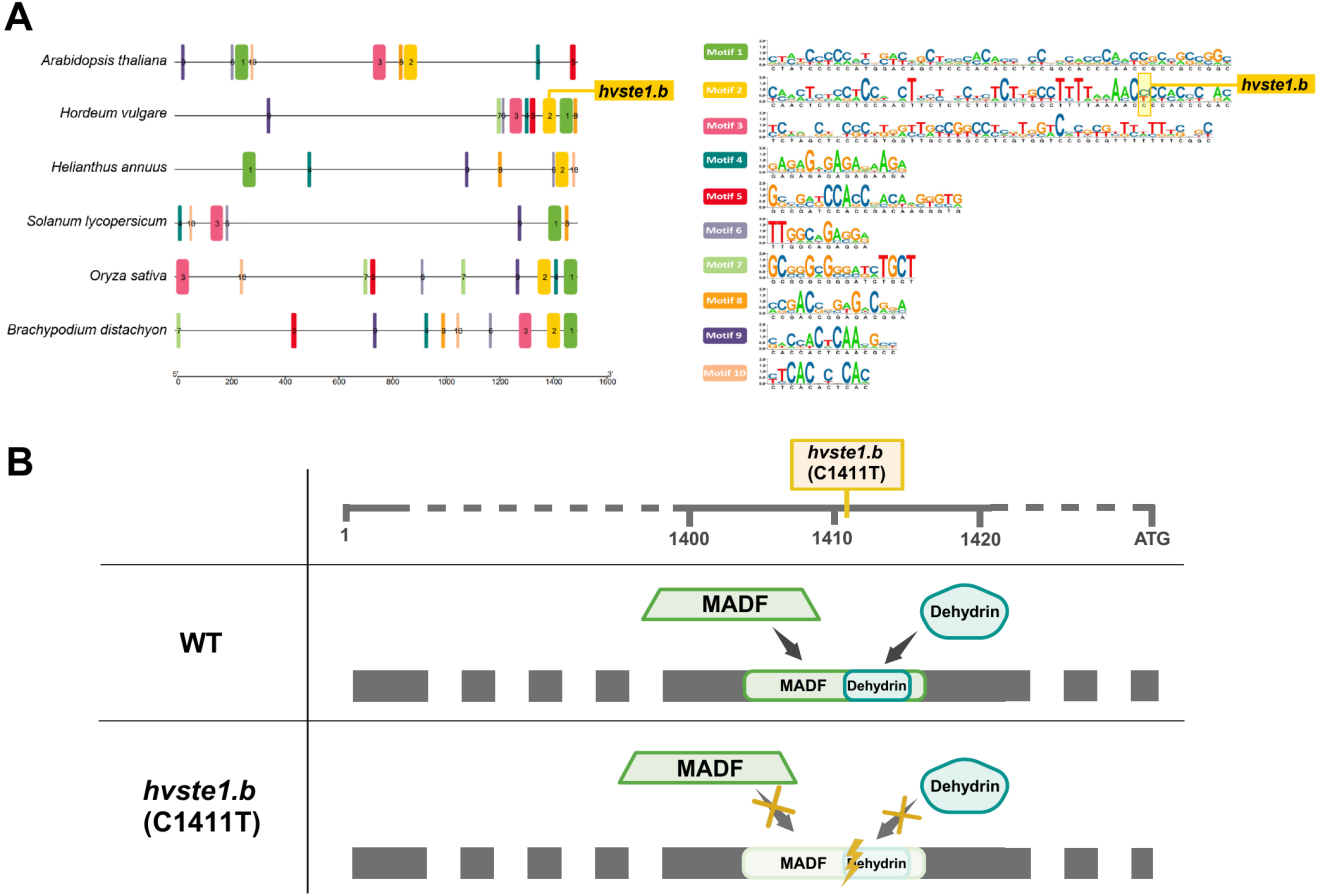
Bioinformatics analysis of *HvSTE1* promoter region and its orthologs. **(A)** *In silico* analysis of the promoter regions of the *STE1* genes and the occurrence of highly conserved motifs among orthologous promoter sequences from *Arabidopsis thaliana*, *Hordeum vulgare, Helianthus annuus, Solanum lycopersicum, Oryza sativa* and *Brachypodium distachyon.* **(B)** Prediction of the *hvste1.b* mutation impact on the occurrence of transcription factors-binding sites. The upper horizontal line represents simplified promoter region of the *HvSTE1* gene with marked position of the *hvste1.b* mutation.

To gain an insight into the regulatory mechanism underlying the decreased expression of the *HvSTE1* gene which resulted from the *hvste1.b* mutation, the *in silico* analysis was performed on the promoter regions of the *STE1* genes. Interestingly, on the basis of analysis of conserved motifs, it was indicated that the *hvste1.b* mutation is located within highly conserved motif (Motif 2) which is present in five out of six promoters of homologous *STE1* genes from the analyzed monocots and dicots species (**Figure 7A**). Noteworthy, out of the identified ten conserved motifs, three were relatively long (48–50 bp in length), whereas the rest contained 11–21 bp. From the evolutionary point of view, conservation of the long motifs is more significant than the short ones, taking into account the probability of mutation accumulation which is correlated with the sequence length. The *hvste1.b* mutation is located within one of the long conserved motifs (**Figure 7A**) what may indicate that in this case the mutation disrupted the motif which plays a significant role in regulation of the *HvSTE1* gene expression. Furthermore, the promoter region of *HvSTE1* was also analyzed in terms of transcription factor binding sites (TFbs). Importantly, the *hvste1.b* mutation was located within the TFbs recognized by the MADF transcription factors and dehydrins. Importantly, MADF and Dehydrin are the only transcription factors capable of binding to the promoter region which is affected by the *hvste1.b* mutation (**Figure 7B**). Based on the bioinformatics analysis, it was suggested that the *hvste1.b* mutation may preclude recognition of these TFbs by the MADF transcription factors and dehydrins which may explain the decreased expression of the *HvSTE1* gene in this mutant.

### 3.5 Influence of the *hvste1.o* mutation on structure and functionality of the HvSTE1 enzyme

**Figure 8:**
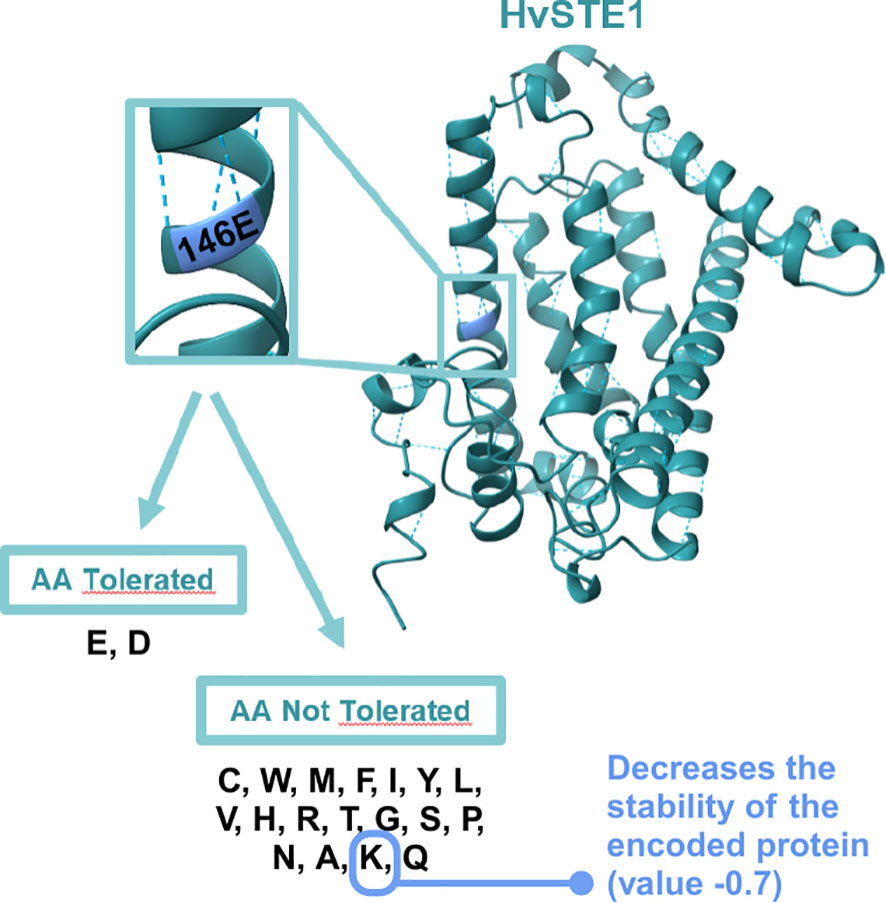
The 3D structure of HvSTE1 protein with the position of E146 residue enlarged and framed and the list of amino acids tolerated and not tolerated at this position. The E146K substitution is not tolerated and causes the significant decrease in stability of the mutated version of HvSTE1.

As far as the *hvste1.o* mutation is concerned, the multiple sequence alignment indicated that the substituted glutamic acid (E146) residue is highly conserved across the analyzed monocot and dicot plant species (**Supplementary Figure S1**). Furthermore, the analysis with the use of I-Mutant3.0 tool (https://folding.biofold.org/i-mutant/) confirmed that the E146K substitution decreases the stability of the encoded protein (value -0.7 indicated significantly decreased stability), and according to the analysis performed with the SIFT tool the E146K mutation affects HvSTE1 functionality (score 0.03). Interestingly, the analysis indicated that at the 146 position only one amino acid (aspartic acid, D), in addition to the glutamic acid (E) which is present in the wild-type form of HvSTE1, is predicted to be tolerated without altering the protein activity (**Figure 8**). These analyses indicated that the glutamic acid (E146) residue, which was substituted in the *hvste1.o* mutant, may have a crucial role in the HvSTE1 protein functionality (**Supplementary Figure S3**). These results may also explain the profound effect of the *hvste1.o* allele on the severity of phenotype of the mutant plants. We analyzed the HvSTE1 protein sequence with the ProtScale program in order to determine the hydropathicity/Kyte Doolittle plot and predict the transmembrane tendency. Both analyses indicated that the E146 residue is localized within one of the hydrophobic, transmembrane domains (**Figure 1B**). Our further analysis of the HvSTE1 protein sequence using the NCBI database and the InterPro program indicated that the E146 residue is localized in the proximal part of the fatty acid hydroxylase functional domain pfam04116 (location of the functional domain within the protein sequence: 138-268, according to NCBI, and 139–268 according to InterPro) (**Figure 1B**).

## 4 Discussion

Our previous *in silico* analyses of the barley genome assembly (*Hordeum vulgare* IBSC_v2) using the Ensembl Plants database, which preceded the identification and functional analysis of the *HvSTE1* gene, indicated that this gene was present in the telomeric region on chromosome 3 as a singleton which means it is not duplicated. This observation was important for the further functional analysis of this gene, as it eliminated a potential problem of gene function redundancy during interpretation of effects of the identified mutations.

Analysis of promoter region of the *STE1* gene has never been carried out in any plant species. Therefore, our aim was to elucidate the effect of the *hvste1.b* allele, particularly because it led to the semi-dwarf phenotype of the *hvste1.b* mutant plants, and this phenotype is of particular importance in cereal breeding (Chono et al., 2003; Sakamoto et al., 2006; Dockter et al., 2014). Furthermore, it is also important to note that the *hvste1.b* mutation did not cause any negative effect on the tiller number (in fact, it caused a slight increase in this parameter) and spike length (**Figures 2B, C**). Both these traits are recognized as very important agronomic traits in cereal breeding (Yang et al., 2024). On the other hand, this mutation resulted in the significant shortening of awns (**Figure 2D**) which is considered, apart from the plant height reduction, as a typical feature of the BR-related mutants in barley (Dockter et al., 2014). Thus, it may be inferred that the phenotype of the *hvste1.b* mutant is caused specifically by the abnormalities in the BR homeostasis. As far as other traits of agronomic importance are concerned, it was observed that in the *hvste1.b* mutant the grain length was slightly decreased when compared with the reference variety ‘Sebastian’, whereas grain width and thickness remained unchanged (**Figures 3A–D**). Interestingly, it was also observed that the 100-grain weight in this mutant was slightly (5%) higher than in the ‘Sebastian’ variety (**Figure 3E**). It is an important result, as it is known that the grain size and weight are crucial agronomic parameters in cereal breeding (Sadras, 2007; Fan and Li, 2019). It should be also kept in mind that several previously identified cereal mutants defective in the BR biosynthesis showed yield decrease, including grain/kernel size reduction, as the side effect of the disturbances in the BR homeostasis (Mori et al., 2002; Hong et al., 2005; Makarevitch et al., 2012; Wu et al., 2016; Zhou et al., 2017; Huang et al., 2022; Xu et al., 2022; Zhang et al., 2024). Thus, it may be concluded that some of the phenotypic traits (semi-dwarfism and shorter awns) exhibited by the *hvste1.b* mutant (**Figures 2A, D**) are typical for the BR-deficient mutants of barley (Dockter et al., 2014), whereas other phenotypic features of this mutant (including the relevant agronomic traits, such as tiller number, spike length, and 100-grain weight) remained unchanged or even slightly improved when compared with the reference variety (**Figures 2B, C**, **3E**). As far as the tiller number is concerned, it should also be mentioned that it was decreased in several previously identified BR-deficient, cereal mutants (Mori et al., 2002; Best et al., 2016; Zhou et al., 2017; Huang et al., 2022). Noteworthy, shortening of spikes was observed in previously identified BR mutants of barley, including the *uzu* mutant which had constituted a source of semi-dwarfism in barley breeding in East Asia (Chono et al., 2003; Dockter et al., 2014).

Since the mutation identified in the *hvste1.b* allele is located in the regulatory region of the *HvSTE1* gene, the first step was to determine the effect of this mutation on expression profile of this gene. In order to analyze the effect of the *hvste1.b* mutation on the gene expression, further analysis with the application of the RT-qPCR method was performed on the 3rd internode and mature embryos (**Figures 5A, D**). In a separate RT-qPCR experiment, we observed that the *HvSTE1* gene expression is much lower (below detection level) in leaves and roots of 8-day-old barley seedlings of the analyzed genotypes. A relatively high expression level of the *HvSTE1* homolog - *OsDWF7* in rice embryo and lower expression in rice seedlings has been recently reported (Zebosi et al., 2024). However, it should be mentioned that in the study by Zebosi et al. (2024) the *OsDWF7* expression was not analyzed in the internodes. The RT-qPCR analysis performed in our study indicated that the *HvSTE1* gene expression was significantly (500-fold) decreased in the 3rd internode of the *hvste1.b* mutant when compared with the reference variety ‘Sebastian’ (**Figure 6A**). As it is known that the major role of BRs is stimulation of plant growth through enhancement of cell division and elongation (Tong and Chu, 2018; Nolan et al., 2020), we postulated that such significant decrease in the *HvSTE1* gene expression in the 3rd internode may be a causal factor for the semi-dwarf phenotype of the *hvste1.b* mutant. Noteworthy, the analysis of the *HvSTE1* gene expression in the mature embryos indicated that in the *hvste1.b* mutant the expression is lower (by ca. 27%) when compared with the ‘Sebastian’ variety (**Figure 6B**). This relatively lower difference in the gene expression in the embryos (when compared with the significant difference which was observed in the 3rd internode) may explain the above-mentioned mild effect of the *hvste1.b* allele on grain size (**Figure 3**). The *hvste1.b* mutation is located in one of the highly conserved motif 2 which is present in promoter regions of homologous genes from dicot and monocot species (**Figure 7A**). Moreover, the *hvste1.b* mutation is located in the promoter region containing binding sites for two transcription factors MADF and Dehydrin. Our *in silico* analysis indicated that the mutation eliminates both transcription factor-binding sites (**Figure 7B**), and it may be responsible for the lowered expression of the *HvSTE1* gene in the *hvste1.b* mutant. However, we could not find any specific information regarding a role of the MADF transcription factor in regulation of BR biosynthesis-dependent plant development in the Plant Transcription Factor Database (PlantTFDB 4.0, https://ngdc.cncb.ac.cn/databasecommons/database/id/307; accessed on the 22nd of August, 2024). Nevertheless, it is known that a group of MADS-box transcription factors plays diverse roles in regulation of the BR signaling and leaf inclination in rice (Duan et al., 2006; Lee et al., 2008). Since, there was no previous information about an involvement of the MADS-box transcription factors in regulation of expression of the BR biosynthesis genes in any plant species, the data obtained in this study opened a new field for further research. Plant dehydrins belong to the most important cellular players involved mostly in responses to various stress conditions (Szlachtowska and Rurek, 2023). However, we have not found any previous report on an involvement of dehydrins in the regulation of BR biosynthesis. Therefore, the results of this study provided a novel insight into this aspect. Finally, in our study the endogenous BR quantification verified that the *hvste1.b* mutation located within the binding sites for the MADF and dehydrin transcription factors causes the decrease in the *HvSTE1* gene expression (**Figure 6**), and influences accumulation of BL. Therefore, this study provided a direct link between the location of this mutation in the *HvSTE1* promoter region, decreased expression of this BR biosynthesis gene (particularly in the 3rd internode), the decrease in the BL accumulation, and consequently, the semi-dwarf phenotype of the *hvste1.b* mutant.

The second mutation (*hvste1.o*) which had the profound effect on the plant phenotype resulted in the E146K substitution (**Figure 1A**; **Table 1**). This allele caused severe dwarfism of the mutant plants, as well as significant reduction in tiller number, spike length, and awn length (**Figure 2**). These phenotypic features represent a typical ideotype of the BR-deficient cereal mutants (Mori et al., 2002; Dockter et al., 2014; Best et al., 2016; Zhou et al., 2017; Huang et al., 2022). Apart from these traits, the *hvste1.o* mutant plants exhibited complete sterility (**Figure 2F**). In order to confirm these aspects of the *hvste1.o* mutant phenotype (and due to sterility of this mutant) we developed the homozygous *hvste1.o* mutant plants from M_2_ heterozygotes in several different attempts. In each case the identified homozygous *hvste1.o* mutant plants exhibited the above-mentioned phenotype (including the complete sterility). These results indicated that the E146 residue is crucial for the enzymatic activity of the HvSTE1 Δ(7)-sterol-C5-desaturase. The *in silico* analysis of the HvSTE1 protein showed that the E146K substitution causes a significant decrease in stability of the mutant version of the HvSTE1 protein. Moreover, another analysis indicated that at the E146 position there is only one tolerated substitution (E146D), whereas all other substitutions (including E146K) are not tolerated and cause defects in the protein activity (**Figure 8**). Indeed, the E146 residue is highly conserved among homologous STE1 proteins from monocot and dicot species (**Supplementary Figure S1**). Apparently, the E146 residue is crucial for the function of the HvSTE1 enzyme. The E146 residue is localized within one of the hydrophobic, transmembrane domains (**Figure 1B**). Analyses of the STE1 protein in Arabidopsis also indicated that the corresponding E140 residue is located within one of the transmembrane domains (Choe et al., 1999), close to the end of a hydrophobic segment and in proximity of one of the conserved histidine clusters (Husselstein et al., 1999). Importantly, the E146 residue is localized in the proximal part of the fatty acid hydroxylase functional domain pfam04116 (**Figure 1B**). Therefore, the E146 residue is located at the junction of two aforementioned domains – structural (transmembrane) and functional (pfam04116). It should be also kept in mind that from the biochemical point of view, in the *hvste1.o* mutant the negatively charged E146 (glutamic acid) residue was substituted by positively charged (lysine) residue what potentially may change the conformation and function of the *hvste1.o* mutant version of the protein. The above results indicated that the *hvste1.o* mutation may have a significant, negative effect on the function of the HvSTE1 Δ(7)-sterol-C5-desaturase as the BR biosynthesis enzyme. Indeed, the BL content in the *hvste1.o* mutant was profoundly decreased when compared with the ‘Sebastian’ variety – it was even lower than in the *hvste1.b* mutant (**Figure 4**). This result may explain the severity of the *hvste1.o* mutant phenotype (including complete sterility). Noteworthy, nonsense mutations of the *STE1* gene in Arabidopsis also led to plant height reduction and decreased fertility (Choe et al., 1999). Thus, we concluded that the significantly decreased BL accumulation observed in the *hvste1.o* mutant stems from profound dysfunction of the HvSTE1 enzyme which results from the substitution of the highly conserved, negatively charged glutamic acid residue, which is located in the hydrophobic segment in proximity of the conserved histidine cluster within the fatty acid hydroxylase functional domain, by positively charged lysine residue. Moreover, our analysis of gene ontology indicated that some of the functions performed by HvSTE1 are carried out by a relatively limited group of proteins (**Supplementary Figure S4**). It may explain the fact that the HvSTE1 enzyme dysfunction results in the severe phenotype due to lack (or low level) of functional redundancy during the progress of these processes.

## 5 Conclusions

Our current understanding of the BR biosynthesis (particularly its early phase) and regulation of this process in cereal crops is still limited. In this study, the *HvSTE1* gene was identified and functionally analyzed in barley which is an important cereal crop. Effects of the identified alleles on the *HvSTE1* gene expression, sequence of the encoded enzyme variants, the BR accumulation, as well as on stature, agronomic traits, and reproduction of the identified mutants were characterized.

Homozygous mutants carrying two mutations (*hvste1.b* and *hvste1.o*) displayed reduced plant height and defects in the BR accumulation. The *HvSTE1* expression was considerably decreased in the 3rd internode of the *hvste1.b* mutant. The *HvSTE1* expression decline resulted from the substitution located in the 5’UTR region of this gene. Moreover, the *hvste1.b* mutant plants showed semi-dwarf phenotype without any negative effect on crucial agronomic traits, such as tiller number, spike length, and grain weight. Therefore, the *hvste1.b* allele allows for achieving a balance between the favorable alteration in plant architecture (semi-dwarfism) and maintenance of grain weight in barley.

On the other hand, the *hvste1.o* mutant displayed severe dwarf phenotype and produced significantly lower number of tillers. Moreover, the *hvste1.o* mutant plants were completely sterile, due to the fact that their inflorescences were underdeveloped. The severe changes in the phenotype of this mutant indicated that the *HvSTE1* gene plays an important role in the BR biosynthesis-dependent regulation of the important agronomic traits in barley, i.e. plant stature, tiller number, and inflorescence development. However, the results of this study indicated that fine-tuning of the *HvSTE1* gene function may allow for achieving the balance between the favorable alteration in plant architecture (semi-dwarfism) and maintenance of grain weight in barley. Therefore, this study provided a novel insight into the function of the *HvSTE1* gene in the BR biosynthesis-dependent regulation of architecture and reproduction of barley being a cereal crop species of crucial importance for the global agriculture.

## Data Availability

The original contributions presented in the study are included in the article/**Supplementary Material**. Further inquiries can be directed to the corresponding author.
