# Supplementary material for: STay tunEd: mutational analysis of the HvSTE1 gene in barley provides insight into the balance between semi-dwarfism and maintenance of grain size in brassinosteroid biosynthesis-dependent manner
Source: Front Plant Sci. 2025 May 6;16:1571368. doi: 10.3389/fpls.2025.1571368 (PMC12089960; doi:10.3389/fpls.2025.1571368)
Supplement: Supplementary file 1 [file DataSheet1.pdf]

Supplementary Material

Supplementary Table S1. Details of primer pairs STE1_1F_1R and STE1_2F_2R which were applied for amplification of the first and the second exons of the *HvSTE1* gene with the aim of mutations occurrence confirmation.

Primer Name	Primer Sequence	GC%	Tm (°C)
**STE1_1F_1R**	F: 5’ GGCATTTGTGTCACGCTCT 3’	52,6	64,0
	R: 5’ CCTCACCAACAAATCGACCT 3’	50,0	63,9
**STE1_2F_2R**	F: 5’ GTTTCCCCCTTTTCCTGTTC 3’	50,0	63,4
	R: 5’CATCCCAGAGGAACAAAGCTA 3’	47,6	63,0

Supplementary Table S2. PCR profiles for the STE1_1F_1R and STE1_2F_2R primers.

	Temperature [°C]		Duration	No. of cycles
	**STE1_1F_1R**	**STE1_2F_2R**		
Initial denaturation	95°C	95°C	5 mins	1
Denaturation	95°C	95°C	45 sec	3
Annealing	66°C	61°C	50 sec	
Extension	72°C	72°C	1 min 30 sec	
Denaturation	95°C	95°C	45 sec	3
Annealing	64°C	59°C	50 sec	
Extension	72°C	72°C	1 min 30 sec	
Denaturation	95°C	95°C	45 sec	36
Annealing	62°C	57°C	50 sec	
Extension	72°C	72°C	1 min 30 sec	
Final extension	72°C	72°C	5 mins	1
Pause	15°C	15°C	Pause	1

Supplementary Table S3. Details of primer pairs STE1_B, EF1, and H2A which were used with the aim of *HvSTE1* gene expression analysis.

Primer Name	Primer Sequence	GC%	Tm (°C)
**STE1_B**	F: 5’ AGACGGACTGGTACAACGAGA 3’	52	57
	R: 5’ AGATGACGAAGCACCAGAGG 3’	55	57
**EF1**	F: 5’ CCCTCCTCTTGGTCGTTTTG 3’	55	58
	R: 5’ ATGACACCAACAGCCACAGTTT 3’	45	58
**H2A**	F: 5’ AGCGTTTAGCTGTGCTCCTTCC 3’	54	58
	R: 5’ TGACTCAATCGGTACCAGGGAAAC 3’	50	58

Supplementary Table S4. RT-PCR profile for the STE1_B, EF1, and H2A primer pair.

	Temperature [°C]	Duration	No. of cycles
Initial denaturation	95°C	3 mins	1
Denaturation	95°C	45 sec	
Annealing	63°C	45 sec	4
Extension	72°C	1 min	
Denaturation	95°C	45 sec	
Annealing	61°C	45 sec	4
Extension	72°C	1 min	
Denaturation	95°C	45 sec	
Annealing	57/58°C	45 sec	36
Extension	72°C	1 min	
Final extension	72°C	5 mins	1
Pause	8°C	Pause	1

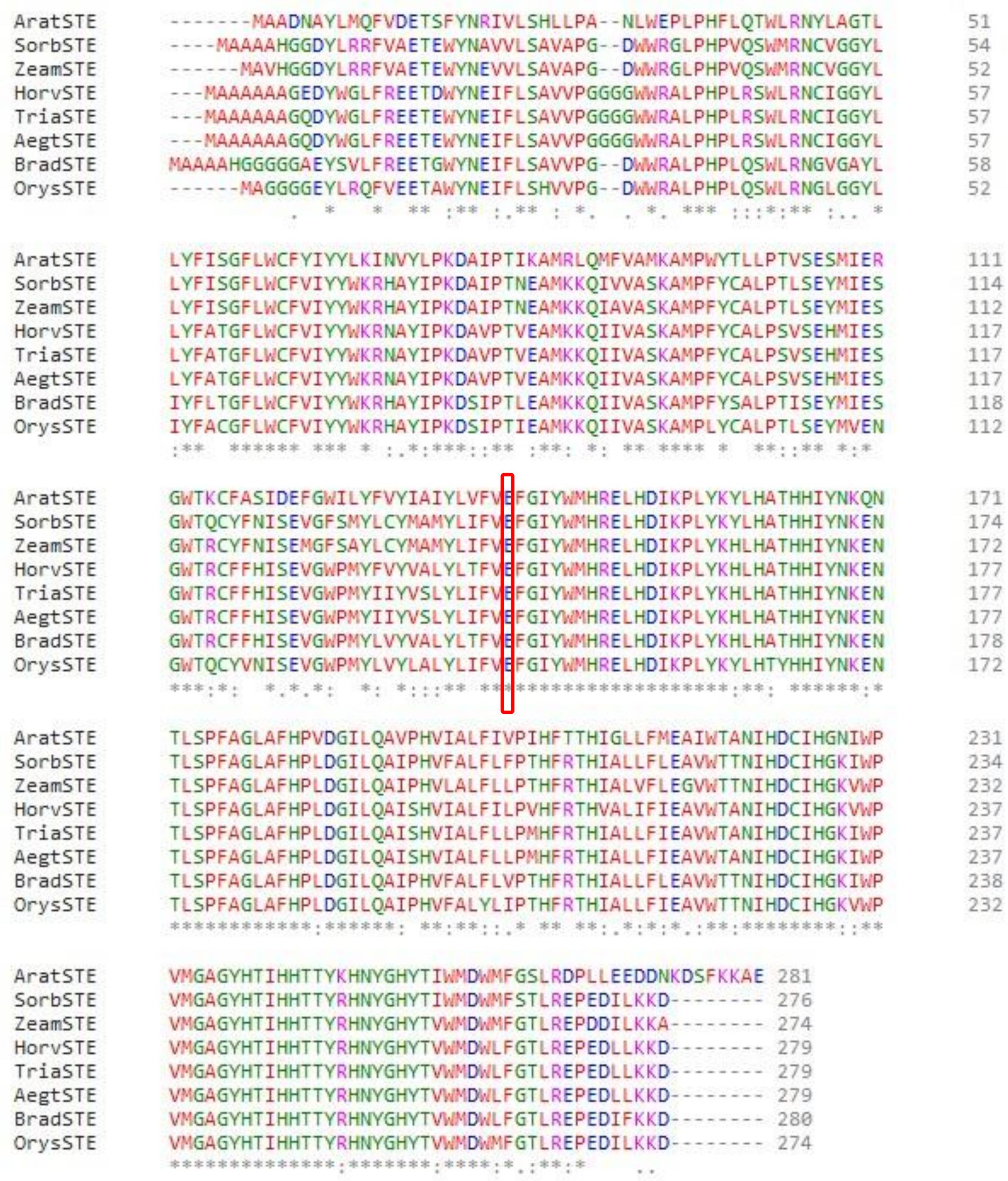

Supplementary Fig. S1. The multiple sequence alignment (MSA) of the STE1 proteins with the use of Clustal Omega tool. The position of substituted amino acid (E146K, allele *hvste1.o*) as a result of the identified mutation is indicated by the red frame. Arat - *Arabidopsis thaliana*, Sorb - *Sorghum bicolor*, Zeam - *Zea mays*, Horv - *Hordeum vulgare*, Tria - *Triticum aestivum*, Aegt - *Aegilops tauschii*, Brad - *Brachypodium distachyon*, Orys - *Oryza sativa*.

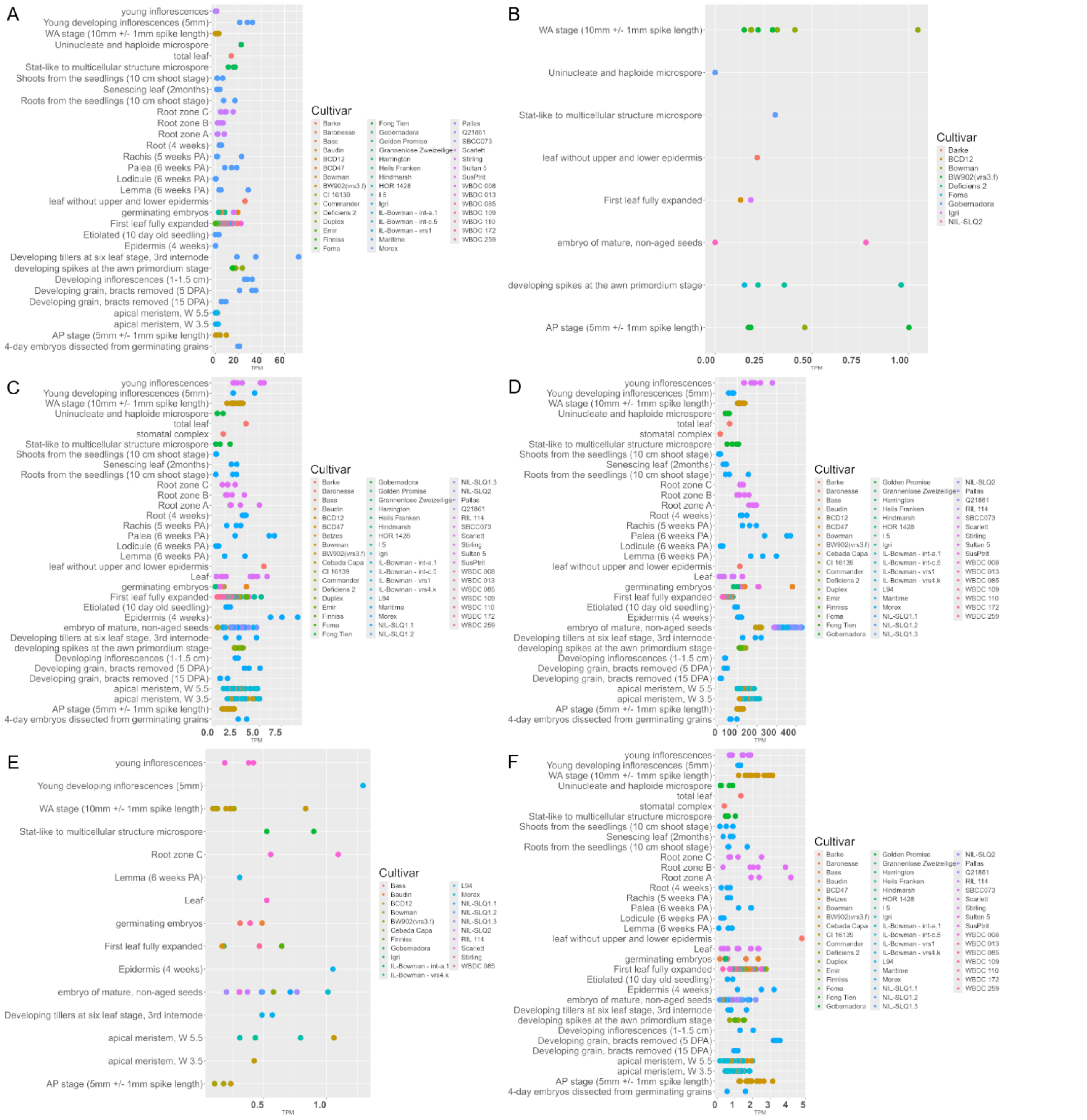

Supplementary Fig. S2. Tissue-specific expression of six *HvSTE1* transcript variants (A - BART1_0-u17568.001, B - BART1_0-u17568.002, C - BART1_0-u17568.003, D - BART1_0-u17568.004, E - BART1_0-u17568.005, F - BART1_0-u17568.006) in various tissues and organs of different barley cultivars (TPM - transcript per million). The expression data for these transcripts in various tissues were retrieved from the EoRNA database.

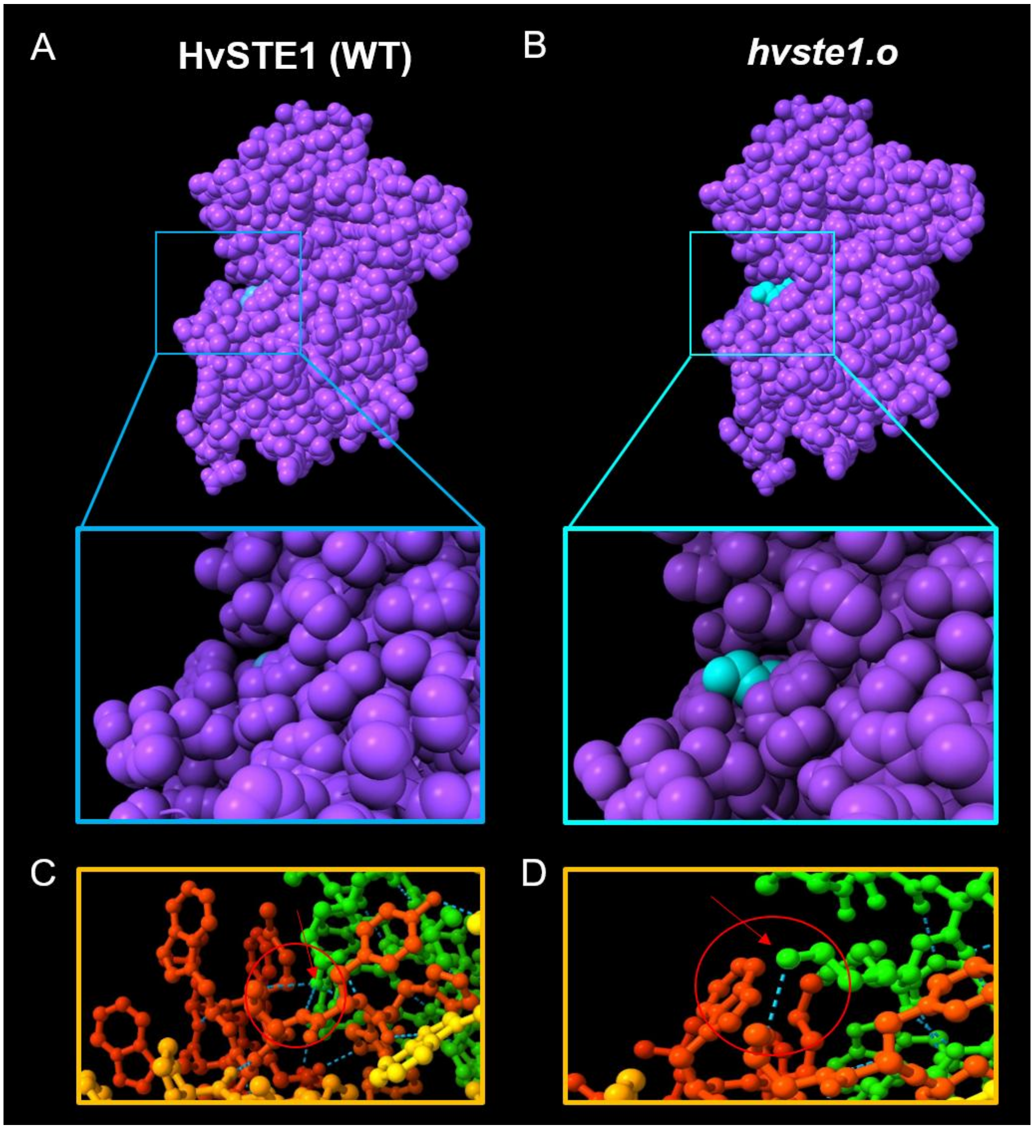

Supplementary Fig. S3. Visualization of the 3D structures and surfaces of HvSTE1 (A) and the mutated version of this protein (B) predicted based on the AlphaFold server and UCSF ChimeraX. The localization of the 146 position in each of the structures is indicated by the blue color. (C-D) The localization of the E146 position (C) and E146K substitution (D) (displayed by the red arrows) with atoms in sphere style and rainbow (N to C-terminus) coloring. The changes in hydrogen-bond (blue dashed lines) pattern is indicated by the red oval.

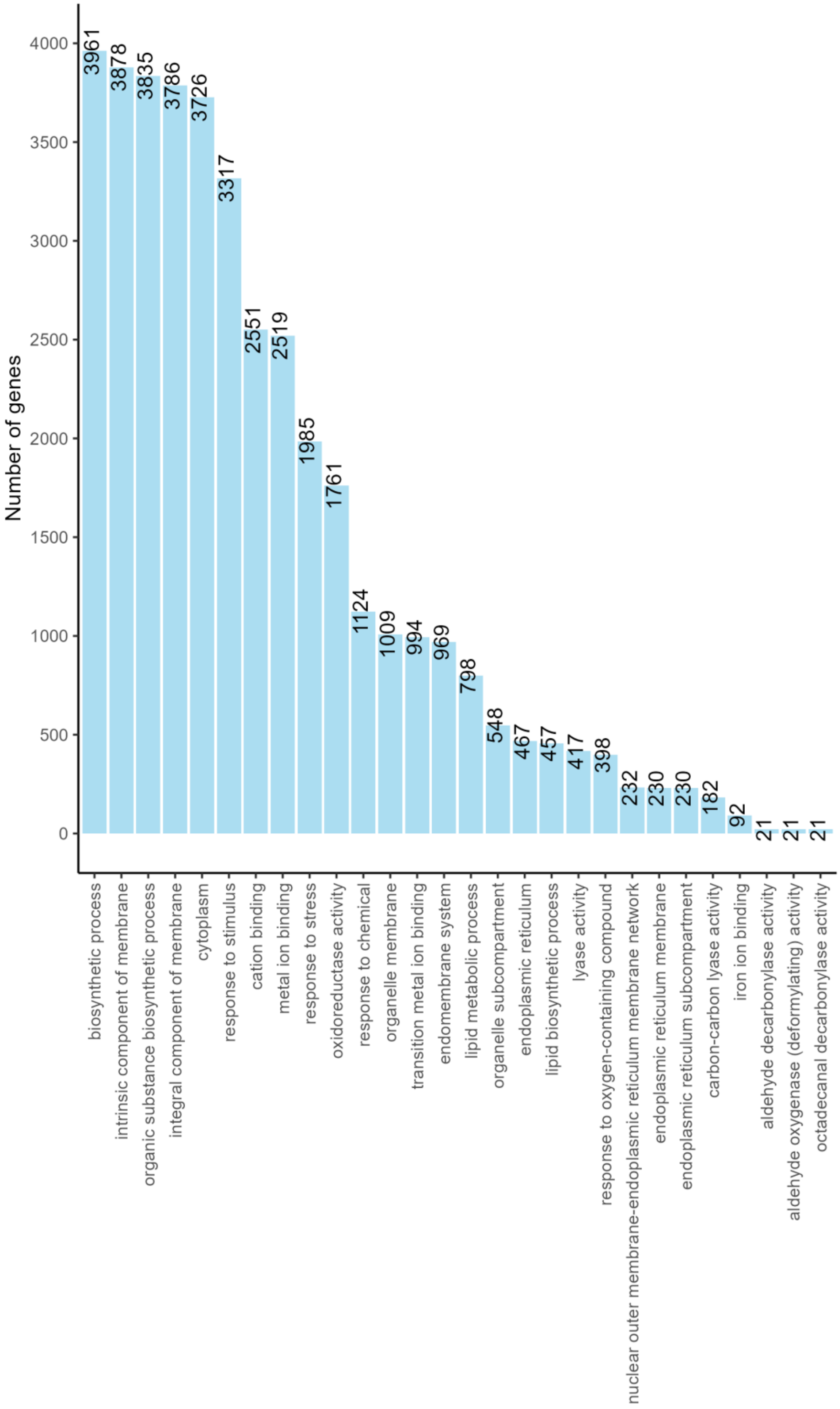

Supplementary Fig. S4. *HvSTE1* gene ontology annotation. The number of genes that take part in each of the biological process is indicated above the bars.
